# Association of the geriatric nutritional risk index with mortality in patients with rheumatoid arthritis: A cohort study from the NHANES 2007–2018

**DOI:** 10.1097/MD.0000000000050312

**Published:** 2026-08-28

**Authors:** Zhong Wen, Shunbing Wang, Yuzhi Li, Xin Tu, Quanbo Zhang, Yufeng Qing

**Affiliations:** a Department of Rheumatology and Immunology, Affiliated Hospital of North Sichuan Medical College, Nanchong, Sichuan, China; b School of Clinical Medicine, North Sichuan Medical College, Nanchong, Sichuan, China; c Research Center of Hyperuricemia and Gout, Affiliated Hospital of North Sichuan Medical College, Nanchong, Sichuan, China; d Department of Geriatrics, Affiliated Hospital of North Sichuan Medical College, Nanchong, Sichuan, China.

**Keywords:** all-cause mortality, cardiovascular mortality, geriatric nutritional risk index, NHANES, rheumatoid arthritis

## Abstract

To investigate the association of the geriatric nutritional risk index (GNRI) with all-cause and cardiovascular mortality among adults with rheumatoid arthritis (RA). Data were obtained from the National Health and Nutrition Examination Survey 2007 to 2018. The GNRI was evaluated continuously and categorically (< 98 vs ≥ 98). Survey-weighted Cox proportional hazards models were used to estimate hazard ratios and 95% confidence intervals (CIs) for all-cause and cardiovascular mortality. Restricted cubic spline, competing-risk, subgroup, and sensitivity analyses were conducted as secondary analyses. Participants with RA had a lower GNRI than those without RA (102.7 vs 105.1; *P* < .001) and higher all-cause (10.0% vs 6.6%) and cardiovascular mortality (2.7% vs 1.5%). In the fully adjusted model, higher GNRI was associated with a lower risk of all-cause mortality (hazard ratio per 1-unit increase, 0.94; 95% CI, 0.89–1.00; *P* = .032). Participants with a GNRI <98 had a higher risk of all-cause mortality than those with a GNRI ≥98 (hazard ratio, 1.84; 95% CI, 1.12–3.04; *P* = .017). Neither continuous nor categorical GNRI was significantly associated with cardiovascular mortality (*P* > .05). A lower GNRI was associated with higher all-cause mortality among participants with RA and may help identify individuals at increased risk. Further prospective studies are needed to determine whether the GNRI provides clinically useful risk information beyond established prognostic factors.

## 1. Introduction

Rheumatoid arthritis (RA) is a chronic systemic autoimmune disease that primarily affects the joints but may also involve extra-articular organs.^[1]^ It is associated with substantial disability and healthcare burden.^[2]^ The 2017 Global Burden of Disease study reported increases of 7.4% in the age-standardized global prevalence of RA and 8.2% in its incidence between 1990 and 2017.^[3]^ Compared with the general population, individuals with RA have been reported to have a 54% higher risk of all-cause mortality^[4]^ and an approximately 50% higher risk of cardiovascular death.^[3]^

Malnutrition is associated with disability, impaired recovery, and higher short- and long-term mortality across chronic diseases,^[5–7]^ as well as poorer quality of life among older adults with chronic conditions.^[8]^ Malnutrition is common among people with RA, although prevalence estimates vary substantially according to the study population and assessment method. A previous study of 1976 United States (U.S.) adults with self-reported RA in National Health and Nutrition Examination Survey (NHANES) 1999 to 2014 reported malnutrition prevalences of 18.8% using the Controlled Nutritional Status score and 26.6% using the Nutritional Risk Index, and both indices were associated with all-cause mortality.^[9]^ Malnutrition in RA may result from systemic inflammation,^[10]^ metabolic disturbances,^[11]^ and disuse atrophy related to reduced physical activity.^[12]^ Rheumatoid cachexia, characterized by weight loss, muscle wasting, protein depletion, and metabolic abnormalities, may further compromise nutritional status.^[13]^ Malnutrition may therefore contribute to adverse outcomes and excess mortality in RA.^[14,15]^

The geriatric nutritional risk index (GNRI) combines serum albumin and the ratio of actual to ideal body weight to estimate nutrition-related risk.^[16]^ It has been validated for predicting nutrition-related complications^[17]^ and mortality in older adults.^[18]^ A cross-sectional study suggested that the GNRI may be associated with disease activity and bone mineral density in patients with RA receiving biologic disease-modifying antirheumatic drugs.^[19]^ However, evidence regarding its association with all-cause and cardiovascular mortality in adults with RA remains limited. We, therefore, examined these associations using linked NHANES and mortality data.

## 2. Methods

### 2.1. Study population

**Figure 1.**
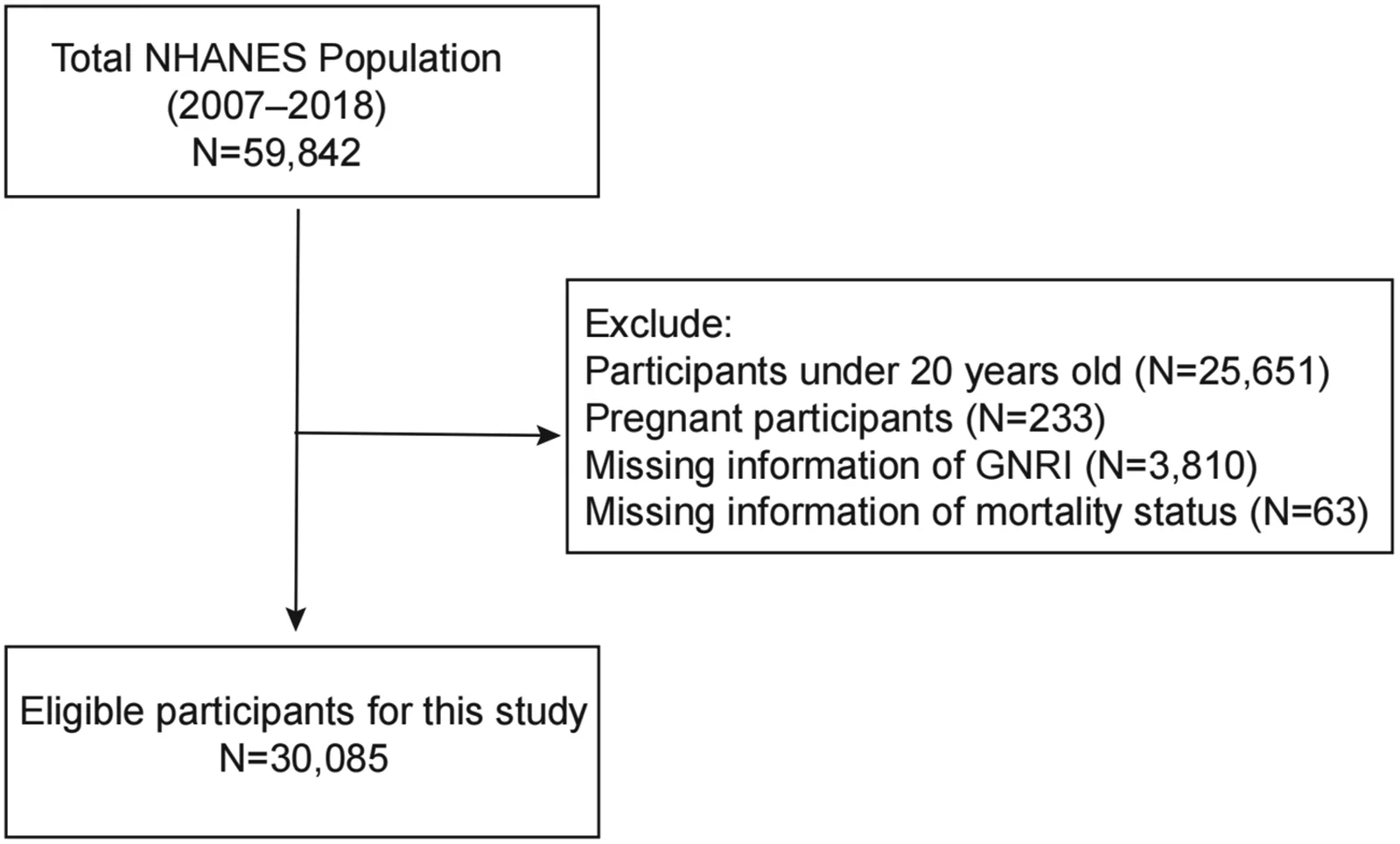
Participant selection flowchart. GNRI = Geriatric Nutritional Risk Index, N = number of participants, NHANES = National Health and Nutrition Examination Survey.

This study used data from NHANES, a survey conducted by the National Center for Health Statistics (NCHS) with a complex, stratified, multistage probability sampling design to represent the U.S. civilian noninstitutionalized population. We pooled data from the 2007 to 2018 survey cycles and linked them to mortality records through December 31, 2019. A total of 30,085 participants met the eligibility criteria, including 999 participants with RA. The participant selection process is shown in Figure 1. The NCHS Ethics Review Board approved the NHANES protocols, and all participants provided informed consent. All procedures were conducted in accordance with the relevant NCHS guidelines and regulations.

### 2.2. Assessment of the GNRI

The GNRI was calculated from each participant’s height (cm), weight (kg), ideal body weight (kg), and serum albumin concentration (g/L), as follows:

GNRI = 1.489 × serum albumin (g/L) + 41.7 × (actual weight/ideal body weight).

Ideal body weight was calculated as follows:

For men: height − 100 − [(height − 150)/ 4]

For women: height − 100 − [(height − 150)/ 2.5]

If actual body weight exceeded ideal body weight, the ratio of actual to ideal body weight was set to 1. Participants were categorized into a high-GNRI group (GNRI ≥ 98) and a low-GNRI group (GNRI < 98).

### 2.3. Assessment of RA

Participants were first asked, “Has a doctor or other health professional ever told you that you had arthritis?” Those who answered “Yes” were then asked to identify the type of arthritis from the following options: “RA,” “Osteoarthritis,” “Psoriatic arthritis,” “Other,” “Refused,” or “Do not know.”^[20]^

### 2.4. Mortality determination

Mortality status was ascertained by linkage to the NCHS National Death Index, with follow-up through December 31, 2019. Causes of death were classified according to the Tenth Revision of the International Classification of Diseases (https://icd.who.int/browse10/2019/en#/IX). All-cause mortality included death from any cause. Cardiovascular mortality was defined using Tenth Revision of the International Classification of Diseases codes I00–I09, I11, I13, I20–I51, and I60–I69 (20).

### 2.5. Covariate assessment

Covariates included sociodemographic characteristics, lifestyle factors, and health conditions. Sociodemographic variables comprised age, sex, race and ethnicity, marital status, educational attainment, and family income. Lifestyle variables included alcohol consumption, smoking status, and physical activity. Body mass index was categorized as underweight (< 18.5 kg/m^2^), normal weight (18.5 to < 25 kg/m^2^), overweight (25 to < 30 kg/m^2^), or obesity (≥ 30 kg/m^2^). Medical history included hypertension, diabetes, cancer, and cardiovascular disease (CVD), defined as coronary heart disease, stroke, or heart failure. CVD history was obtained through computer-assisted interviews. Hypertension was defined as systolic blood pressure ≥ 140 mm Hg, diastolic blood pressure ≥ 90 mm Hg, or reported use of antihypertensive medication.^[21]^ Diabetes was defined as fasting plasma glucose ≥ 7.0 mmol/L, random plasma glucose ≥ 11.1 mmol/L, glycated hemoglobin ≥ 6.5%, current use of antidiabetic medication, or a physician diagnosis.^[22]^ Laboratory covariates included the neutrophil-to-lymphocyte ratio, a marker of systemic inflammation.^[23]^

### 2.6. Statistical analysis

The NHANES complex sampling design was accounted for using the Mobile Examination Center (MEC) examination weights, masked variance pseudo-strata (SDMVSTRA), and pseudo-primary sampling units (SDMVPSU). Because six 2-year NHANES cycles from 2007–2018 were combined, a 12-year MEC examination weight was calculated by dividing the 2-year MEC examination weight (WTMEC2YR) by 6. A survey design object was constructed using the survey package in R with nest = TRUE. Analyses restricted to participants with RA were conducted as domain analyses by subsetting the survey design object after the full design had been specified. Strata containing a single primary sampling unit (PSU) were handled by centering the lonely PSU at the overall mean (survey.lonely.psu = “adjust”), and the same adjustment was applied to domain-specific lonely PSUs (survey.adjust.domain.lonely = TRUE).

Unweighted sample sizes and survey-weighted descriptive estimates are reported. Continuous variables are presented as survey-weighted medians with interquartile ranges and were compared using design-based Wilcoxon rank-sum tests. Categorical variables are presented as unweighted counts with survey-weighted percentages and were compared using second-order Rao–Scott *F*-adjusted chi-square tests.

As a secondary comparison, participants with and without RA were matched 1:1 using nearest-neighbor propensity score matching (PSM) without replacement, with a caliper of 0.2 standard deviations of the logit of the propensity score. Propensity scores were estimated using age, sex, and race and ethnicity. Covariate balance after matching was evaluated using standardized mean differences, with an absolute standardized mean difference < 0.10 indicating acceptable balance. The propensity score-matched sample was used only to compare participants with and without RA and was not used in the mortality analyses.

Among participants with RA, survey-weighted Cox proportional hazards models were fitted to estimate hazard ratios (HRs) and 95% confidence intervals (CIs) for the associations of the GNRI with all-cause and cardiovascular mortality. Potential nonlinear associations were examined by incorporating restricted cubic spline (RCS) terms into the survey-weighted Cox models. Three knots were placed at the 10th, 50th, and 90th percentiles of the GNRI distribution.^[24]^ Survey-weighted Kaplan–Meier curves were generated according to GNRI category, and survival distributions were compared using design-based log-rank tests. A Fine–Gray subdistribution hazards model was additionally fitted as a secondary exploratory analysis of cardiovascular mortality, with noncardiovascular death treated as the competing event and the GNRI modeled as a continuous variable. Subgroup analyses evaluated the association between GNRI category and all-cause mortality across age, sex, race and ethnicity, family income, and prespecified clinical strata. Interaction terms between GNRI category and each subgroup variable were tested in separate survey-weighted Cox models. Sensitivity analyses were performed after restricting the sample to participants aged ≥ 60 years. Analyses were performed using R version 4.3.1 (R Core Team, R Foundation for Statistical Computing). All tests were 2-sided, and *P* values < .05 were considered statistically significant.

## 3. Results

### 3.1. Participant baseline characteristics

The analysis included 30,085 participants: 29,086 without RA (weighted n = 201,098,646) and 999 with RA (weighted n = 5,510,630). Participants with RA were older than those without RA (median age, 59 vs 47 years) and were more often women (59% vs 51%). Most participants with RA were non-Hispanic White (63%) and married (55%). Compared with participants without RA, those with RA had lower educational attainment and family income, a higher prevalence of obesity (48%), and a lower prevalence of normal weight (20%). Sitting time was similar between groups, but fewer participants with RA met physical activity recommendations, and more were inactive. Participants with RA also had higher prevalences of hypertension (62%), diabetes (24%), cancer (17%), and CVD. The median GNRI was lower in participants with RA than in those without RA (102.7 vs 105.1; *P* < .001). All-cause mortality (10.0% vs 6.6%) and cardiovascular mortality (2.7% vs 1.5%) were also higher among participants with RA (Table 1).

**Table 1:** Baseline characteristics of NHANES participants (2007–2018).

Characteristic	Overall	Non-RA	RA	*P*
	N = 30,085	N = 29,086	N = 999	
Age (yr)	48 (34–61)	47 (34–60)	59 (50–68)	< .001
Age group				< .001
< 60 years	19,722 (73)	19,315 (74)	407 (52)	
60–69 years	4878 (13)	4583 (13)	295 (23)	
70–79 years	2967 (7.6%)	2810 (7.4)	157 (13)	
80+ years	2518 (6.1)	2378 (6.0)	140 (12)	
Sex				.004
Female	15,403 (51)	14,817 (51)	586 (59)	
Male	14,682 (49)	14,269 (49)	413 (41)	
Race				.006
Mexican American	4570 (8.5)	4425 (8.5)	145 (8.6)	
Other Hispanic	3194 (5.9)	3088 (5.9)	106 (5.7)	
Non-Hispanic White	12,468 (67)	12,115 (67)	353 (63)	
Non-Hispanic Black	6172 (11)	5870 (10)	302 (16)	
Other/multiracial	3681 (8.0)	3588 (8.0)	93 (7.3)	
Marital status				< .001
Married	15,701 (56)	15,214 (56)	487 (55)	
Never married	5140 (17)	5056 (17)	84 (7.3)	
Living with partner	2409 (8.0)	2352 (8.1)	57 (5.7)	
Other	6835 (19)	6464 (18)	371 (32)	
Education attainment				< .001
High school or less	14,260 (39)	13,716 (38)	544 (48)	
Some College	8778 (31)	8450 (31)	328 (36)	
College Graduate or above	7047 (30)	6920 (31)	127 (15)	
Family income				< .001
High income	5401 (27)	5289 (27)	112 (17)	
Low income	10,748 (25)	10,280 (25)	468 (36)	
Medium income	13,936 (48)	13,517 (48)	419 (47)	
Alcohol consumption				< .001
Nondrinker	8752 (23)	8388 (23)	364 (31)	
1–5 drinks/month	14,760 (49)	14,276 (49)	484 (49)	
5–10 drinks/month	2321 (9.4)	2273 (9.5)	48 (5.2)	
10+ drinks/month	4252 (18)	4149 (18)	103 (14)	
Smoking status				< .001
Never smoker	16,673 (55)	16,195 (56)	478 (45)	
Former smoker	7308 (25)	7011 (25)	297 (31)	
Current smoker	6104 (20)	5880 (20)	224 (24)	
BMI (kg/m^2^)	28 (24–33)	28 (24–33)	30 (25–35)	< .001
BMI group (kg/m^2^)				< .001
Normal (18.5 to < 25)	8005 (27)	7830 (28)	175 (20)	
Obese (30 or greater)	11,653 (38)	11,157 (38)	496 (48)	
Overweight (25 to < 30)	9976 (33)	9659 (33)	317 (31)	
Underweight (< 18.5)	451 (1.4)	440 (1.4)	11 (1.1)	
MET (min/wk)	1440 (180–4320)	1440 (180–4320)	720 (0–3600)	< .001
MET group (min/wk)				< .001
Sufficient PA	17,494 (63)	16,999 (63)	495 (53)	
Insufficient PA	3848 (13)	3726 (13)	122 (12)	
Low PA	644 (1.9)	622 (1.9)	22 (1.8)	
Inactive PA	8099 (22)	7739 (22)	360 (33)	
GNRI	104.7 (101.3–108.7)	105.1 (101.3–108.7)	102.7 (99.8–105.7)	< .001
NLR	1.96 (1.50–2.60)	1.96 (1.50–2.60)	2.03 (1.50–2.76)	.1
Hypertension				< .001
Yes	13,287 (39)	12,600 (38)	687 (62)	
No	16,798 (61)	16,486 (62)	312 (38)	
Diabetes				< .001
Yes	5585 (14)	5257 (14)	328 (24)	
No	24,500 (86)	23,829 (86)	671 (76)	
Congestive heart failure				< .001
Yes	991 (2.4)	894 (2.2)	97 (8.2)	
No	29,094 (98)	28,192 (98)	902 (92)	
Coronary heart disease				< .001
Yes	1279 (3.6)	1192 (3.5)	87 (8.2)	
No	28,806 (96)	27,894 (97)	912 (92)	
Angina pectoris				< .001
Yes	769 (2.2)	711 (2.1)	58 (6.3)	
No	29,316 (98)	28,375 (98)	941 (94)	
Stroke				< .001
Yes	1158 (2.9)	1067 (2.7)	91 (8.3)	
No	28,927 (97)	28,019 (97)	908 (92)	

Data are represented as n (%).

BMI = body mass index, GNRI = geriatric nutritional risk index, MET = metabolic equivalent, PA = physical activity, RA = rheumatoid arthritis, N = number of participants, NHANES = National Health and Nutrition Examination Survey, NLR = neutrophil-to-lymphocyte ratio.

PSM yielded 983 matched pairs of participants with and without RA; 16 participants with RA could not be matched (Table 2). After PSM, no statistically significant differences were observed in age group, sex, race and ethnicity, marital status, educational attainment, family income, alcohol consumption, smoking status, body mass index category, sitting time, or physical activity between the 2 groups. The median GNRI remained lower among participants with RA than among those without RA (102.75 [interquartile range, 99.77–105.73] vs 104.24 [interquartile range, 99.77–107.22]; *P* = .002). Most examined comorbidities were also comparable between the matched groups, although congestive heart failure remained more frequent among participants with RA (8.6% vs 6.0%; *P* = .030).

**Table 2:** Baseline characteristics of participants stratified by RA after propensity score matching, NHANES 2007–2018.

Characteristic	Overall N = 1966	Non-RA N = 983	RA N = 983	*P*
Age group				.520
< 60 years	797 (40.5)	391 (39.8)	406 (41.3)	
60+ years	1169 (59.5)	592 (60.2)	577 (58.7)	
Sex				.463
Female	1163 (59.2)	590 (60.0)	573 (58.3)	
Male	803 (40.8)	393 (40.0)	410 (41.7)	
Race				.928
Mexican American	294 (15.0)	149 (15.2)	145 (14.8)	
Other Hispanic	204 (10.4)	99 (10.1)	105 (10.7)	
Non-Hispanic White	713 (36.3)	362 (36.8)	351 (35.7)	
Non-Hispanic Black	564 (28.7)	275 (28.0)	289 (29.4)	
Other/multiracial	191 (9.7)	98 (10.0)	93 (9.5)	
Marital status				.858
Married	974 (49.5)	489 (49.7)	485 (49.3)	
Never married	173 (8.8)	89 (9.1)	84 (8.5)	
Living with partner	106 (5.4)	49 (5.0)	57 (5.8)	
Other	713 (36.3)	356 (36.2)	357 (36.3)	
Education attainment				.618
High school or less	1091 (55.5)	556 (56.6)	535 (54.4)	
Some College	630 (32.0)	309 (31.4)	321 (32.7)	
College Graduate or above	245 (12.5)	118 (12.0)	127 (12.9)	
Family income				.597
High income	252 (12.8)	128 (13.0)	124 (12.6)	
Low income	938 (47.7)	478 (48.6)	460 (46.8)	
Medium income	776 (39.5)	377 (38.4)	399 (40.6)	
Alcohol consumption				.673
Nondrinker	729 (37.1)	369 (37.5)	360 (36.6)	
1–5 drinks/month	950 (48.3)	477 (48.5)	473 (48.1)	
5–10 drinks/month	87 (4.4)	38 (3.9)	49 (5.0)	
10+ drinks/month	200 (10.2)	99 (10.1)	101 (10.3)	
Smoking status				.929
Never smoker	947 (48.2)	472 (48.0)	475 (48.3)	
Former smoker	578 (29.4)	287 (29.2)	291 (29.6)	
Current smoker	441 (22.4)	224 (22.8)	217 (22.1)	
BMI group (kg/m^2^)				.855
Normal (18.5 to < 25)	344 (17.5)	170 (17.3)	174 (17.7)	
Obese (30 or greater)	965 (49.1)	480 (48.8)	485 (49.3)	
Overweight (25 to < 30)	633 (32.2)	319 (32.5)	314 (31.9)	
Underweight (< 18.5)	24 (1.2)	14 (1.4)	10 (1.0)	
MET group (min/wk)				.389
Sufficient PA	958 (48.7)	469 (47.7)	489 (49.7)	
Insufficient PA	262 (13.3)	141 (14.3)	121 (12.3)	
Low PA	49 (2.5)	28 (2.8)	21 (2.1)	
Inactive PA	697 (35.5)	345 (35.1)	352 (35.8)	
GNRI	102.75 (99.77–105.73)	104.24 (99.77–107.22)	102.75 (99.77–105.73)	.002
NLR	2.00 (1.47–2.76)	2.00 (1.48–2.70)	2.00 (1.45–2.80)	.716
Hypertension				.304
Yes	1364 (69.4)	693 (70.5)	671 (68.3)	
No	602 (30.6)	290 (29.5)	312 (31.7)	
Diabetes				.382
Yes	617 (31.4)	299 (30.4)	318 (32.3)	
No	1349 (68.6)	684 (69.6)	665 (67.7)	
Congestive heart failure				.030
Yes	144 (7.3)	59 (6.0)	85 (8.6)	
No	1822 (92.7)	924 (94.0)	898 (91.4)	
Coronary heart disease				.239
Yes	153 (7.8)	69 (7.0)	84 (8.5)	
No	1813 (92.2)	914 (93.0)	899 (91.5)	
Angina pectoris				.36
Yes	102 (5.2)	46 (4.7)	56 (5.7)	
No	1864 (94.8)	937 (95.3)	927 (94.3)	
Stroke				.156
Yes	156 (7.9)	69 (7.0)	87 (8.9)	
No	1810 (92.1)	914 (93.0)	896 (91.1)	

Data are represented as n (%).

BMI = body mass index, GNRI = geriatric nutritional risk index, MET = metabolic equivalent, PA = physical activity, RA = rheumatoid arthritis, N = number of participants, NHANES = National Health and Nutrition Examination Survey, NLR = neutrophil-to-lymphocyte ratio.

Baseline characteristics by GNRI category among participants with RA are presented in Table 3. The low-GNRI group was older (median age, 61 years vs 58 years; *P* = .022) and included a higher proportion of women (72% vs 57%; *P* = .026). Its median GNRI was 95.3, compared with 104.2 in the high-GNRI group (*P* < .001). Non-Hispanic White participants constituted the largest racial and ethnic group (55%). Physical inactivity was numerically more common in the low-GNRI group than in the high-GNRI group (40% vs 32%), although the overall difference in physical activity categories was not statistically significant (*P* = .066). The low-GNRI group had a higher neutrophil-to-lymphocyte ratio (2.38 vs 2.00; *P* = .046). No statistically significant between-group differences were observed for hypertension or diabetes.

**Table 3:** Baseline characteristics of participants stratified by GNRI, NHANES 2007 to 2018.

Characteristic	Overall N = 999 (100%)	High-GNRI (GNRI ≥ 98) N = 842 (87%)	Low-GNRI (GNRI < 98) N = 157 (13%)	*P*
Age (yr)	59 (50–68)	58 (49–68)	61 (51–71)	.022
Age group				.12
< 60 years	407 (52)	356 (54)	51 (39)	
60–69 years	295 (23)	244 (22)	51 (31)	
70–79 years	157 (13)	129 (13)	28 (16)	
80+ years	140 (12)	113 (12)	27 (14)	
Sex				.026
Female	586 (59)	484 (57)	102 (72)	
Male	413 (41)	358 (43)	55 (28)	
Race				.032
Mexican American	145 (8.6)	130 (8.8)	15 (7.2)	
Other Hispanic	106 (5.7)	98 (6.0)	8 (3.8)	
Non-Hispanic White	353 (63)	304 (64)	49 (55)	
Non-Hispanic Black	302 (16)	237 (15)	65 (24)	
Other/multiracial	93 (7.3)	73 (6.9)	20 (9.9)	
Marital status				.6
Married	487 (55)	421 (55)	66 (53)	
Never married	84 (7.3)	66 (7.0)	18 (8.8)	
Living with partner	57 (5.7)	52 (6.1)	5 (3.5)	
Other	371 (32)	303 (32)	68 (35)	
Education attainment				.12
High school or less	544 (48)	455 (47)	89 (58)	
Some College	328 (36)	276 (38)	52 (25)	
College Graduate or above	127 (15)	111 (15)	16 (17)	
Family income				.057
High income	124 (21)	103 (20)	21 (29)	
Low income	472 (36)	392 (35)	80 (41)	
Medium income	403 (43)	347 (45)	56 (30)	
Alcohol consumption				.058
Nondrinker	367 (32)	297 (29)	70 (46)	
1–5 drinks/month	482 (47)	409 (48)	73 (41)	
5–10 drinks/month	49 (5.5)	43 (5.4)	6 (6.0)	
10+ drinks/month	101 (16)	93 (17)	8 (6.9)	
Smoking status				.2
Never smoker	478 (45)	418 (46)	60 (37)	
Former smoker	297 (31)	248 (31)	49 (30)	
Current smoker	224 (24)	176 (23)	48 (32)	
BMI (kg/m^2^)	30 (25–35)	29 (26–34)	31 (24–38)	.4
BMI group (kg/m^2^)				.004
Normal (18.5 to < 25)	175 (20)	139 (20)	36 (24)	
Obese (30 or greater)	496 (48)	416 (47)	80 (56)	
Overweight (25 to < 30)	317 (31)	283 (33)	34 (16)	
Underweight (< 18.5)	11 (1.1)	4 (0.7)	7 (3.8)	
MET (min/wk)	720 (0–3600)	720 (0–3840)	360 (0–2880)	.056
MET group (min/wk)				.066
Sufficient PA	495 (53)	420 (54)	75 (47)	
Insufficient PA	122 (12)	105 (13)	17 (8.3)	
Low PA	22 (1.8)	15 (1.4)	7 (4.5)	
Inactive PA	360 (33)	302 (32)	58 (40)	
GNRI	102.7 (99.8–105.7)	104.2 (101.3–107.2)	95.3 (93.8–96.8)	< .001
NLR	2.04 (1.50–2.77)	2.00 (1.50–2.70)	2.38 (1.53–3.30)	.046
Hypertension				.078
Yes	687 (62)	570 (61)	117 (73)	
No	312 (38)	272 (39)	40 (27)	
Diabetes				.058
Yes	328 (24)	265 (23)	63 (33)	
No	671 (76)	577 (77)	94 (67)	
Congestive heart failure				.3
Yes	97 (8.2)	75 (7.9)	22 (10)	
No	902 (92)	767 (92)	135 (90)	
Coronary heart disease				.071
Yes	88 (8.3)	68 (7.5)	20 (14)	
No	911 (92)	774 (92)	137 (86)	
Angina pectoris				.7
Yes	58 (6.3)	48 (6.1)	10 (7.4)	
No	941 (94)	794 (94)	147 (93)	
Stroke				.3
Yes	91 (8.3)	69 (8.0)	22 (11)	
No	908 (92)	773 (92)	135 (89)	

Data are represented as n (%).

BMI = body mass index, GNRI = geriatric nutritional risk index, MET = metabolic equivalent, PA = physical activity, RA = rheumatoid arthritis, N = number of participants, NHANES = National Health and Nutrition Examination Survey, NLR = neutrophil-to-lymphocyte ratio.

### 3.2. Analysis of the association between the GNRI and mortality in RA

Cox proportional hazards models evaluated the GNRI as both a continuous and a categorical variable (Table 4). In Model 3, higher GNRI was significantly associated with a lower risk of all-cause mortality (HR per 1-unit increase, 0.94; 95% CI, 0.89–1.00; *P* = .032). Participants with a GNRI < 98 had a significantly higher risk of all-cause mortality than those with a GNRI ≥ 98 (HR, 1.84; 95% CI, 1.12–3.04; *P* = .017). Neither continuous nor categorical GNRI was significantly associated with cardiovascular mortality.

**Table 4:** Association of GNRI with all-cause and cardiovascular mortality among participants with RA.

Characteristic	Model 1		Model 2		Model 3	
	HR (95% CI)	*P*	HR (95% CI)	*P*	HR (95% CI)	*P*
All-cause mortality						
GNRI	0.94 (0.89–0.99)	.011	0.94 (0.89–0.99)	.017	0.94 (0.89–1.00)	.032
GNRI categorical						
≥ 98	Reference		Reference		Reference	
< 98	2.02 (1.06–3.87)	.033	1.90 (1.01–3.57)	.045	1.84 (1.12–3.04)	.017
Cardiovascular mortality						
GNRI	0.96 (0.88–1.06)	.4	0.97 (0.89–1.07)	.6	0.99 (0.89–1.10)	.9
GNRI categorical						
≥ 98	Reference		Reference		Reference	
< 98	1.41 (0.51–3.88)	.5	1.15 (0.46–2.85)	.8	0.91 (0.36–2.28)	.8

Model 1: unadjusted model; Model 2: adjusted for age, sex, and race; Model 3: fully adjusted model, including additional covariates such as marital status, education attainment, family income, alcohol consumption, smoking status, hypertension, and diabetes.

CI = confidence interval, GNRI = geriatric nutritional risk index, HR = hazard ratio, RA = rheumatoid arthritis.

**Figure 2.**
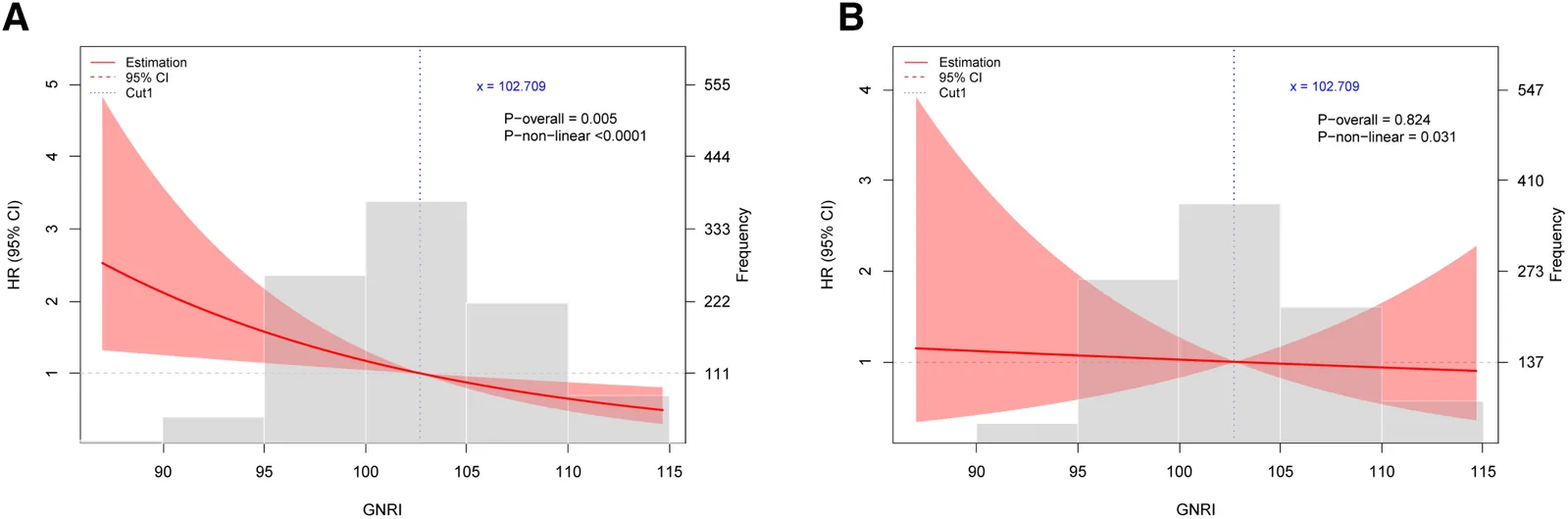
Restricted cubic spline analysis of the associations of the GNRI with all-cause and cardiovascular mortality in RA. (A) Association between the GNRI and all-cause mortality; (B) association between the GNRI and cardiovascular mortality. CI = confidence interval, GNRI = Geriatric Nutritional Risk Index, HR = hazard ratio, RA = rheumatoid arthritis.

A 3-knot RCS model was used to characterize the association between the GNRI and mortality. Knots were placed at the 10th, 50th, and 90th percentiles of the GNRI distribution. The RCS analysis showed that the estimated hazard of all-cause mortality generally decreased with increasing GNRI, particularly at lower GNRI values (*P* < .0001; Fig. 2A). With a GNRI of 102.709 as the reference, the estimated hazard was higher at lower GNRI values. For cardiovascular mortality, neither the overall association nor the nonlinear component was statistically significant (*P* > .05; Fig. 2B).

**Figure 3.**
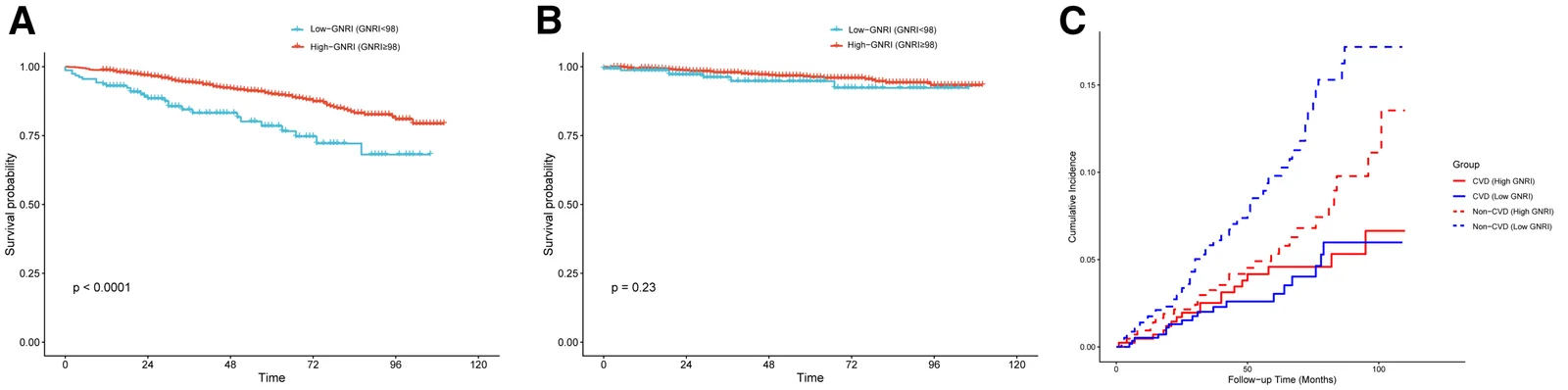
Kaplan–Meier survival curves and cumulative incidence of mortality among participants with RA according to GNRI category. (A) Kaplan–Meier curve for all-cause mortality; (B) Kaplan–Meier curve for cardiovascular mortality; (C) cumulative incidence functions for cardiovascular and noncardiovascular mortality, with noncardiovascular death treated as the competing event for cardiovascular mortality. CVD = cardiovascular disease, GNRI = Geriatric Nutritional Risk Index, RA = rheumatoid arthritis.

Kaplan–Meier curves according to GNRI category are shown in Figure 3. Overall survival was lower in the low-GNRI group than in the high-GNRI group (*P* < .001; Fig. 3A), with a more pronounced decline after 50 months of follow-up. Cardiovascular mortality-free survival did not differ significantly between the groups (Fig. 3B). With noncardiovascular death treated as a competing event, continuous GNRI was not associated with cardiovascular mortality (subdistribution hazard ratio, 0.96; 95% CI, 0.91–1.02; *P* = .218). The cumulative incidence curves suggested a higher incidence of noncardiovascular death in the low-GNRI group, whereas the cardiovascular mortality curves were broadly similar between the GNRI groups (Fig. 3C).

### 3.3. Subgroup analysis and sensitivity analysis

**Figure 4.**
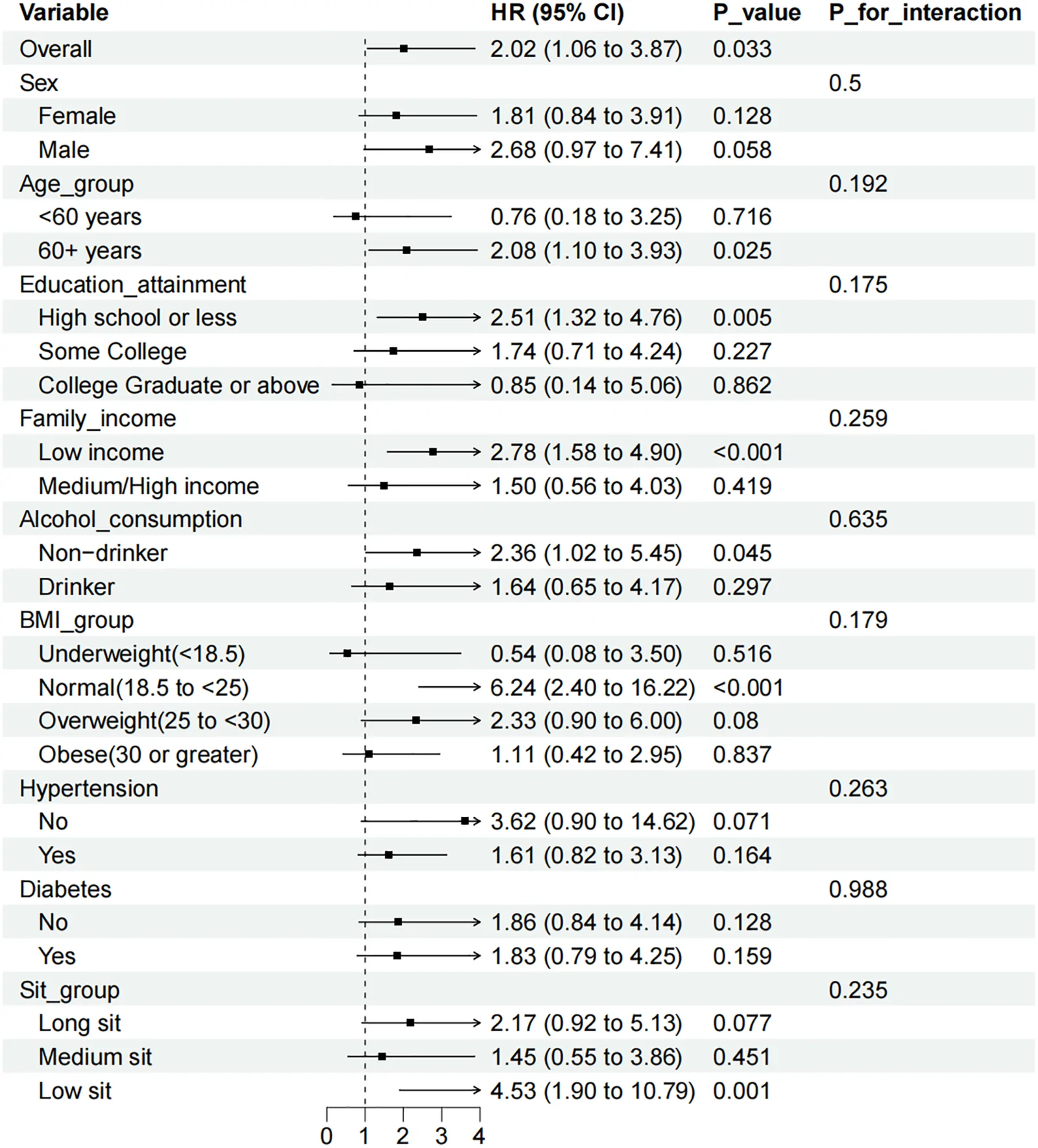
Association between the GNRI and all-cause mortality in subgroups. BMI = body mass index, CI = confidence interval, GNRI = Geriatric Nutritional Risk Index, HR = hazard ratio.

Subgroup analyses were conducted to assess the consistency of the association between GNRI category and all-cause mortality across prespecified participant characteristics (Fig. 4). In the unadjusted overall analysis, participants with a GNRI < 98 had a higher risk of all-cause mortality than those with a GNRI ≥ 98 (HR, 2.02; 95% CI, 1.06–3.87; *P* = .033). Although the magnitude of the association varied and the point estimates were > 1.0 in most strata, none of the interaction tests was statistically significant (all *P* for interaction > .05), indicating no evidence of effect modification by the examined subgroup variables. The sensitivity analysis restricted to participants aged ≥ 60 years yielded a directionally consistent association (Supplemental Table 1, Supplemental Digital Content 1, Supplemental Digital Content, Supplemental Digital Content 1).

## 4. Discussion

Participants with RA were older and more often women, married, obese, and physically inactive than those without RA. They also had higher prevalences of hypertension, diabetes, cancer, and CVD, and higher all-cause and cardiovascular mortality. The median GNRI was lower in participants with RA than in those without RA (102.7 vs 105.1; *P* < .001), and the difference persisted after PSM. This finding suggests a greater burden of nutrition-related risk among participants with RA. It is consistent with a multicenter cross-sectional study in which approximately one-third of older patients with RA had impaired nutritional status associated with older age, greater disease activity, and poorer quality of life.^[15]^ Patients with RA may also have lower serum albumin concentrations than the general population; hypoalbuminemia may reflect systemic inflammation, nutritional impairment, or disease progression.^[25,26]^

Older adults with RA are susceptible to extra-articular manifestations, loss of appetite, sarcopenia, weakness, fatigue, and neuropsychiatric conditions that may contribute to frailty.^[15]^ Akner et al reported that protein–energy malnutrition is common in chronic inflammatory diseases and may worsen disease activity, joint damage, and functional disability in RA. Impaired immune function and greater susceptibility to infection may partly explain the associated increase in mortality risk.^[27]^

Participants in the low-GNRI group tended to be older, were more often women, and had higher prevalences of obesity and physical inactivity than those in the high-GNRI group. RA affects women more frequently than men, with an approximate female-to-male ratio of 3:1,^[28]^ and older patients often have a greater comorbidity burden and poorer overall health.^[29]^ Among older adults with RA, impaired nutritional status is associated with lower physical activity, poorer physical function, and lower quality of life.^[27]^ Management can be challenging because the potential benefits of targeted therapy must be balanced against treatment-related risks in patients with multiple comorbidities.^[29]^ Obesity may further contribute to inflammation through adipose-derived cytokines and adipokines,^[25]^ and sarcopenic obesity is associated with greater inflammatory activity and poorer outcomes in RA.^[30]^

In fully adjusted analyses, higher GNRI was significantly associated with a lower risk of all-cause mortality. This inverse association was supported by the RCS and Kaplan-Meier analyses and remained directionally consistent in subgroup and sensitivity analyses. No statistically significant interaction was detected across the prespecified subgroups. By contrast, the GNRI was not significantly associated with cardiovascular mortality, possibly because of the limited number of cardiovascular deaths and insufficient statistical power.

The GNRI reflects overall nutritional status and has been associated with mortality in several clinical populations. A previous NHANES 1999 to 2014 study included 1976 adults with self-reported RA and evaluated malnutrition using the Controlled Nutritional Status score and the Nutritional Risk Index; both measures were associated with all-cause mortality.^[9]^ The present study differed by focusing on the GNRI as both a continuous measure and a clinically applied binary category, incorporating more recent survey cycles and mortality follow-up, and evaluating cardiovascular mortality and competing risks. Nevertheless, the overlap in data source and outcome means that our findings should be interpreted as an extension of, rather than entirely independent evidence from, the earlier study. Cardiovascular outcomes in RA are influenced by interacting factors, including systemic inflammation, medication exposure, and comorbidities. Chronic inflammation may promote both malnutrition through increased catabolism and CVD through accelerated atherosclerosis.^[31,32]^ Hypertension, diabetes, and pharmacologic treatment may further modify cardiovascular risk.^[33]^ Larger studies are needed to clarify how nutrition, inflammation, and cardiovascular health jointly influence mortality in RA.

Malnutrition in RA is associated with poorer functional outcomes and shorter life expectancy.^[8]^ Frailty is also associated with higher disease activity, more severe pain, and poorer physical function,^[34]^ which may reduce physiologic reserve and increase vulnerability to adverse outcomes. In addition to inflammation-related mechanisms, nutritional deficiencies may impair tissue repair and wound healing and increase susceptibility to severe infection, an important cause of death in RA.^[35]^

Malnutrition may worsen RA outcomes through inflammation, immune dysfunction, and metabolic disturbance.^[36]^ Chronic inflammation increases catabolism and may lead to protein and energy depletion. In turn, nutritional impairment may compromise immune function and tissue repair, potentially reinforcing inflammation and increasing susceptibility to infection.^[12,37]^ Nutritional assessment, including use of the GNRI, may therefore help identify patients with RA who are at increased risk and may benefit from further evaluation or intervention.^[9,38]^

This study has several limitations. First, RA was identified by self-report and may therefore have been misclassified or underreported. Second, the observational design precludes causal inference. The available phenotypic data also did not support Mendelian randomization or instrumental-variable analyses. Third, NHANES does not provide key RA-specific clinical information, including disease activity and treatment, which limited evaluation of disease severity and therapy-related effects. Residual and unmeasured confounding may remain despite multivariable adjustment. Finally, because the study population was drawn from the U.S., the findings may not be generalizable to other countries or healthcare systems. Future studies should include larger and more diverse cohorts with detailed clinical and treatment data. Interventional studies are also needed to determine whether improving nutritional status reduces mortality.

In clinical practice, the GNRI may provide a simple adjunct to nutritional and prognostic assessment in patients with RA. However, chronic inflammation, medication use, changes in body composition, and muscle wasting may influence both the GNRI and clinical outcomes. The GNRI should therefore be interpreted alongside disease activity, body composition, and functional status rather than used in isolation. These findings support routine nutritional screening and individualized assessment, but interventional studies are needed before specific dietary or treatment strategies can be recommended on the basis of this analysis.

## 5. Conclusion

Participants with RA had poorer nutritional status and a lower GNRI than those without RA. Among participants with RA, a lower GNRI was associated with higher all-cause mortality but not cardiovascular mortality. The GNRI may help identify individuals at increased risk, although prospective and interventional validation is needed before it can guide management.

## Author contributions

**Data curation:** Yuzhi Li, Xin Tu.

**Formal analysis:** Zhong Wen, Shunbing Wang.

**Methodology:** Zhong Wen.

**Validation:** Shunbing Wang, Quanbo Zhang.

**Writing – original draft:** Zhong Wen, Shunbing Wang.

**Writing – review & editing:** Yufeng Qing.


