# Supplementary material for: Association of the geriatric nutritional risk index with mortality in patients with rheumatoid arthritis: A cohort study from the NHANES 2007–2018
Source: Medicine (Baltimore). 2026 Aug 28;105(35):e50312. doi: 10.1097/MD.0000000000050312 (PMC13529182; doi:10.1097/MD.0000000000050312)
Supplement: Supplementary file 1 [file medi-105-e50312-s001.docx]

**Supplementary Table 1** Association of GNRI with all-cause and cardiovascular mortality among participants with RA aged ≥60 years.

| **Characteristic** | **Model 1** | | **Model 2** | | **Model 3** | |
| --- | --- | --- | --- | --- | --- | --- |
|  | **HR(95% CI)** | **P** | **HR(95% CI)** | **P** | **HR(95% CI)** | **P** |
| All-cause mortality | |  |  |  |  |  |
| GNRI | 0.95(0.90, 1.01) | 0.120 | 0.95(0.89, 1.01) | 0.100 | 0.95(0.89, 1.01) | **0.084** |
| GNRI categorical |  |  |  |  |  |  |
| ≥98 | Reference |  | Reference |  | Reference |  |
| ＜98 | 2.08(1.10, 3.93) | **0.025** | 2.32(1.20, 4.49) | **0.012** | 2.19(1.31, 3.67) | **0.003** |
| Cardiovascular mortality | |  |  |  |  |  |
| GNRI | 0.97(0.89, 1.06) | 0.500 | 0.97(0.89, 1.05) | 0.400 | 0.98(0.89, 1.07) | 0.700 |
| GNRI categorical |  |  |  |  |  |  |
| ≥98 | Reference |  | Reference |  | Reference |  |
| ＜98 | 1.13(0.45, 2.83) | 0.800 | 1.15(0.46, 2.86) | 0.800 | 0.90(0.36, 2.28) | 0.800 |
| Abbreviations: GNRI: geriatric nutritional risk index; RA: rheumatoid arthritis; HR: hazard ratio; CI: confidence interval. Model 1: unadjusted model; Model 2: adjusted for sex, race; Model 3: fully adjusted model, including additional covariates such as marital status, education attainment, family income, alcohol consumption, smoking status, hypertension, and diabetes. | | | | | | |
